# Recommendations for a balanced approach to supporting academic integrity: perspectives from a survey of students, faculty, and tutors

**DOI:** 10.1007/s40979-022-00116-x

**Published:** 2022-09-12

**Authors:** Cheryl A. Kier, Cindy Ives

**Affiliations:** Faculty of Humanities and Social Sciences, Athabasca University, 1 University Drive, Athabasca, AB T9S 3A3 Canada

**Keywords:** Academic integrity, Academic misconduct, Higher education, Canada, Student perspectives, Instructor perspectives, Recommendations

## Abstract

Maintaining academic integrity is a growing concern for higher education, increasingly so due to the pivot to remote learning in 2020 caused by the COVID-19 pandemic. We canvassed students, faculty, and tutors at an online Canadian university about their perspectives on academic integrity and misconduct. The survey asked how the university could improve policies concerning issues of academic integrity, how faculty and tutors handled cases of misconduct, about satisfaction with how academic violations were treated, and about the role of students, faculty, and tutors in encouraging academic integrity. As well, we collected suggestions from respondents for reducing cheating, addressing academic misconduct, and general ideas about academic integrity. The distinction between misconduct and integrity was not always clear in their comments. We received responses from 228 students and 73 faculty and tutors, generating hundreds of comments. In this paper we focus only on the answers to open-ended questions. Using content analysis, we categorized the replies into similar threads. After multiple iterations of analysis, we extracted three general recommendation groupings: Policy and Procedures, Compliance and Commitment, and Resources. Based on respondents’ views, we propose a balanced approach to supporting academic integrity. Although we conducted the study pre-COVID-19, the recommendations apply to current and future academic integrity practices in our context and beyond.

## Introduction

Maintaining academic integrity is a growing concern for higher education. Such concerns have increased since the pivot to remote learning caused by the COVID-19 pandemic (Rossiter, 2020). There have been calls for more studies in Canada (Awosoga et al., 2021; Hunter, 2016), as the academic culture here is unique (Stoesz & Los, 2019). Findings and recommendations from studies in other countries may not generalize to Canada. Additionally, Canadian studies about student experiences are limited (Packalen & Rowbotham, 2022). More Canadian research is needed on faculty experiences, evidence-based policies, prevention practices, and faculty skill development (Eaton & Edino, 2018).

We aimed to fill such gaps in the literature by investigating how online students and instructors believe adherence to academic integrity can be improved. To explore these issues, our research question was: *What recommendations do students, faculty, and tutors have for improving academic integrity?* We used an exploratory approach to look for deep understandings of the issues as well as to identify concerns and prevention techniques. Our data revealed information about student and instructor experiences, suggestions for policy, ideas for prevention and for instructor professional development. Thus we address several of the recommendations for research on academic integrity in Canada. Students, faculty, and tutors provided their ideas which we then integrated into recommendations supported by strategies for implementation.

### Background and recent literature

There have been numerous attempts to reduce academic misconduct at institutions of higher education (Stoesz & Yudintseva, 2018). These include tutorials (Benson et al., 2019; Kier, 2019), workshops for students (Penaluna & Ross, 2022) and faculty (Curtis et al., 2021), text-matching software (Belli et al., 2020), and sanctions (Gottardello & Karabag, 2020). While many of these strategies may have helped address issues of misconduct at individual or course levels, it is not clear what students and instructors think about more general institution-wide academic integrity concerns. A recent research method is to ask stakeholders for their viewpoints and experiences to get a broader picture of the issues in specific contexts (Gottardello & Karabag, 2020; Lynch et al., 2021).

For example, Packalen and Rowbotham (2022) conducted focus groups with 44 undergraduate students at a Canadian university, who noted that breaches of academic integrity were more likely if instructors failed to make efforts to reduce cheating. Students suggested instructors could show they care about academic integrity by creating new exams and assignments each year. Many students described tension with instructors who they perceived did not appreciate the burdens students face both inside and outside the academy. The authors gleaned 20 recommendations about what students could do and 34 recommendations about what faculty and administration could do to support academic integrity. However, these researchers did not obtain feedback from faculty or administrators. We build on their work by including faculty, a larger sample of students, and by comparing these suggestions to the literature on academic integrity.

As little is known about responsibilities of post-secondary instructors in preventing and responding to academic integrity breaches (Hamilton & Wolsky, 2022), asking them for their viewpoints is an important step. Gottardello and Karabag (2020) interviewed 82 instructors from six countries about their roles in supporting academic integrity. Many instructors perceived educating and supporting students regarding academic integrity as part of their jobs. This included providing instruction on writing for an academic audience. For some it involved providing clear and detailed guidelines for assignments or giving feedback. Other instructors reported using scare tactics to improve the odds of preventing students from academic misconduct. Overall, the researchers noted the importance of the instructor role in maintaining academic integrity, although they acknowledged the difficulty of this due to heavy workloads.

de Maio et al. (2019) interviewed 26 instructors from four Australian universities. They found participants did not always follow the requirements of institutional policies and procedures when responding to academic integrity breaches. The authors stressed that inconsistencies in approach risked the universities’ reputations and broader perception of the quality of the credentials offered.

Mellar et al. (2018) conducted one of the few studies that asked students, instructors and administrators about the extent of cheating at two universities in Turkey and Bulgaria and how this could be prevented. Instructors believed that there was a problematic amount of cheating. Plagiarism and contract cheating were reported as the biggest issues. The most common suggestion from instructors for prevention was education, although other responses included using technology (e.g., text-matching software, author authentication), improved design of assignments, and harsher sanctions. These instructors tended to blame students for being lazy and administrators for imposing weak sanctions. Both instructors and students acknowledged that insufficient education on what constituted plagiarism and cheating was another cause of academic misconduct. Students added that uninteresting courses and heavy workloads were reasons students cheated. The two administrators who were interviewed expressed concern with security and proctoring of exams, especially online exams.

Gaining stakeholder perspectives is important because this facilitates deep, contextual understanding of the issues, leading to buy-in and compliance. This research approach helps identify what needs to be included in policies and procedures, as well as what resources are needed to ensure effective implementation. Meeting the needs of both students and instructors is essential to creating a culture of academic integrity (McNeill, 2022**).** Our study aimed to contribute to the literature about stakeholder viewpoints on academic misconduct and integrity more generally by analyzing and reporting on open-ended comments provided by students, faculty, and tutors from our Canadian online university. The literature cited above is very recent. Our research pre-dated these studies but is aligned with their goals for contextual understanding.

## Research design and methods

Using an anonymous, confidential online survey approved by our Research Ethics Board and the university’s leadership, we posed a series of questions to a representative selection of students, faculty, and tutors. We did not invite administrators to participate, although some respondents pointed to administrator responsibilities in their comments. Participants included 125 undergraduate and 103 graduate students, and 73 faculty and tutors. The distribution of respondents was representative by discipline, with a higher proportion of females. A low overall response rate (5% students; 18% faculty/ tutors) was consistent with similar studies (Christensen Hughes & McCabe, 2006; Zaza & McKenzie, 2018). “Tutor” in our context refers to individualized study tutors, academic experts, markers, lab instructors, group study and study circle instructors. Most tutors have PhDs in the subject area for which they are responsible. Their role is to support and evaluate student learning. We grouped them with faculty because they have similar instructional roles.​

We reported the quantitative results from the close-ended questions internally. In this paper we focus on open-ended responses. Questions were designed to elicit perspectives and ideas about aspects of academic integrity (Table 1).

**Table 1:** Questions and Number of Responses from Participants

Target Audience	Question	Number of responses
Faculty/ tutor	Do you have any suggestions on how your campus might improve its policies concerning academic integrity or any additional comments you care to make?	33
Faculty/ tutor	What role do you think faculty should play in promoting academic integrity and/or controlling cheating in their courses?	30
Faculty/ tutor	What behaviours do you employ to reduce the opportunity and/or temptation for students to cheat in your courses?	9
Faculty/ tutor	If you were convinced that a student had cheated on a major test or assignment in your course, what (a) does your school's policy require you to do, and (b) would be your most likely reaction?	17
Faculty/ tutor	Have you ever referred a suspected case of cheating to your Program Director, Course Coordinator, Chair, Dean, or anyone else? How satisfied were you with the way the case(s) were handled? Please explain your response	13
Student	What specific changes would you like to see your school take in support of academic integrity? What role should students play in this process?	72
Student	Please use this space for any comments you care to make, or if there is anything else you would like to tell us about the topic of cheating	73

The two authors organized the qualitative responses by question then independently coded them. The coding process resulted in dividing many responses into multiple comments. At first we grouped the responses according to who provided them (student or faculty/ tutor), but later according to which role appeared to be responsible for resolving the issues raised: students, faculty/ tutor, or administration.

Next, we imported the data to NVivo™ and reviewed proposed codes synchronously, negotiating to consensus when necessary. Once we finalized 24 codes, we compared them across areas of responsibility, exploring duplications and contradictions. We repeated the analysis in multiple iterations to refine our thinking and to organize and structure our interpretations about comments from each respondent group. This process helped ensure we agreed about the meaning of the data. We then combined the comments into larger categories comprising key areas of concern.

Similar to the approach described by Zadeh et al. (2019), our content analysis “was both deductive and inductive, with transcripts coded for their content, as well as codes being informed by the previous literature on this topic” (p. 7). We followed Saldaña’s (2020) guidance that “one of the coder’s primary goals is to find these repetitive patterns of action and consistencies in human affairs as documented in the data” (p. 5). Our method was similar to that of Bretag and Mahmud (2016) in that “while informed by the literature and the authors’ own experience, the aim was to allow the themes to emerge from the data in grounded theory style, rather than imposing a preconceived set of ideas on the transcripts” (p. 32). The study fits the characteristics of grounded theory, according to the criteria of being believable, adequate, grounded, and applicable (Rennie et al., 1988).

Design and analysis were semi-emergent as we extracted recommendations from the data. Through the process of writing and coding, we learned what we were thinking. We shared those thoughts with one another which led to changes in our analyses over time and ultimately our final interpretations. Recognizing overlap among the different responsibility groups, we re-organized the comments by suggestions for preventing misconduct. Initially, we identified 97 recommendations, but realized that some of these were broader and more conceptual than others. Many suggestions were specific strategies for implementing recommendations, so we consolidated and re-categorized accordingly. As we reorganized the recommendations thematically into areas of concern, we eliminated further duplications. After a final review and recoding of strategies we ended up with 26 recommendations and 40 strategies from 203 unique comments.

## Study findings and related literature

Our study aimed to learn about respondents’ experiences and perceptions related to cheating, plagiarism and other academic misbehaviours related to the question *What recommendations do students, faculty and tutors have for improving academic integrity?* In this section we present summaries of the comments, relate them to the literature, and provide supporting quotes in accompanying Tables. The section is organized in three subsections illustrating respondents’ voices: (1) Policy and Procedures, (2) Compliance and Commitment, and (3) Resources.

### Policy and procedures

We ended up with 83 comments relating personal experience and opinions to academic integrity policies and procedures, suggesting 10 recommendations and seven strategies. Respondents highlighted the importance of policy that defines acceptable and unacceptable behaviours and clearly describes roles, responsibilities, and procedures for managing cases of suspected plagiarism (Table 2).

**Table 2:** Selected Recommendations, Strategies, and Responses for Policy and Procedures

Recommendations	Strategies	Responses
Ensure clarity in procedures and policy	Provide clear and consistent definition(s) of plagiarism, including what could be considered unintentional in the policy	… on several instances, school official stated that if there was any indication that the student intended to cite the source, student would not be charged with plagiarism (for example no quotations on a lengthy quote- but included citation consistent with paraphrasing
Clarity about to whom to report	Ensure responsibilities and workflow actions are clearly described in policies and procedures	More standardized methods of detection In The Faculty of Social Sciences and Humanities, we have a policy that dictates plagiarized assignments be sent to our AU program director for review and consequences. This works extremely well, and the policy should be applied across the institution
Clarify the tutors’ role and distinguish it from that of the person to whom the tutor reports		Not all tutors are aware of the procedures required when academic misconduct is detected. It’s not solely the tutor’s responsibility to mete out punishment, either since the offense should be brought to the attention of the person in your department that handles all academic offenses. This way, there can be a note made on the student’s file for future reference or if a pattern develops
Clarity regarding consequences	Reconsider the “penalty” of allowing a student found committing academic misconduct to rewrite a paper Mete out harsher penalties	The end result was that the student was permitted to re-write the assignment, which I then had to grade. It felt insufficient given the particular scope of the plagiarism in this specific case Punishments for students who cheat should be very severe Failing to cite someone else’s ideas is of course theft, but using the wording of a CITED source should more properly be regarded as sloppy, lazy writing (and awarded a commensurate grade) than reacted to as a serious crime
Clarity about evidence required	Provide PD for faculty/tutors on what constitutes plagiarism	Cheating in distance education can be hard to prove. As a tutor, I can tell if someone is cheating from regularly dealing with the assignments, but a course coordinator who does not deal with the assignments can be more hesitant to accept that someone is cheating
		I strongly recommend that our univesrity [*sic*] acquire plagiarism software. The primary reason tutors don’t catch plagiarism is that they are too busy, and following up on it creates too much hassle
		Instructors should be better versed in the concept of plagiarism

Participants appealed for clear communication of university-wide definitions of plagiarism and cheating, embedded in policy and aligned with detailed steps to follow in cases of alleged misconduct. They proposed that procedures should identify roles and responsibilities at every level of the organization, and that all stakeholders should have access to guidance and support for following them. Everyone should know where to find and how to apply the policy. Faculty and tutors indicated there are various consequences applied, so there was a need for policy, procedures, and sanctions to be implemented consistently across the university. Underpinning many comments was the principle of ensuring students, faculty, tutors, and administrators understand academic integrity and the implications associated with it.

Studies from multiple countries including Australia (Bretag & Mahmud, 2014), Canada (Awosoga et al., 2021), United Kingdom (Brown & Janssen, 2017), and United States (Denney et al., 2020), document similar concerns. It is common for students to expect to learn about paraphrasing and other writing skills at their post-secondary institutions, while instructors expect that students have already mastered these skills (Peters & Cadieux, 2019). This discrepancy can lead to confusion on both sides, so clear policy can help. Researchers advocate comprehensive academic integrity policies that are strategic (Schoenherr & Williams-Jones, 2011), holistic and multi-layered (Stoesz & Eaton, 2020), and are the product of iterative (Bristor & Burke, 2016) and inclusive (Whitley & Keith-Spiegel, 2001) consultation with all stakeholder groups, including students. Recognizing that institution-wide academic integrity depends on more than policy, McCabe (2005) nevertheless called for a comprehensive review of policy. He encouraged wide stakeholder input supported by acknowledgement of the responsibilities and opportunities that emerge from this process. This literature suggests the process of consultation in policy development can lead to greater common understanding of academic integrity.

Substantive policies that enable instructors to act consistently are important (Gottardello & Karabag, 2020), as they promote credibility for the institution. We turn next to the issues of compliance and what our participants and the literature say about the significance of compliance, consistency, and commitment.

### Compliance and commitment

We ended up with 51 comments related to compliance and commitment. From this we derived six recommendations and 12 strategies. Patterns that emerged involved inconsistent application of policy, lack of support from administration, frustration that little was done to deal with perceived cases of misconduct, and confusion about the nature of individual instructors’ roles to deal with cases on their own and to model academic integrity. Other comments highlighted the importance of students’ compliance. Several respondents believed the university had included sufficient barriers to violations of academic misconduct. One noted

in reality, it is each student who must decide the value of their education. I guess some students may just choose to go to Univesity [*sic*] to get a degree and good marks…, but I would guess that a majority of students go to …University because they really want to better themselves. In such cases, cheating would serve no purpose.

Indeed, studies have found that maturity and personal characteristics such as self-efficiency are associated with avoiding cheating (Jurdi et al., 2011).

Another concern was lack of support from administration, however, participants did not always specify who they meant by “administration” (sometimes it was a dean, program director, vice president, course coordinator, or a staff member from the Office of the Registrar) (Table 3).

**Table 3:** Selected Recommendations, Strategies, and Responses for Resources

Recommendations	Strategies	Responses
Take alleged cases seriously throughout the university	Take tutors’ findings seriously and follow up on cases when these are brought to course coordinators	Students regularly plaragarize [*sic*] papers and coordinators and deans often do not take it seriously
	Ensure that administration (and others) take every case seriously by obtaining full information	I have caught students submitting 100% plagiarized papers or papers clearly written by someone else The [administrator] never supports serious penalties (e.g., suspension) so I have stopped bothering to assess these penalties even when warranted. I simply apply the harshest penalty within my discretion. If it is one of my students, they get a zero and that is the end of it. If it is someone else’s student who is referred to me, I give them a zero in the course and notation on their transcript and that is the end of it no matter how many times they appear before me. Why would I beat my head against that wall when the [administrator] doesn’t give a shit? Deans and higher level management should not be allowed to "dismiss" without thoroughly look [*sic*] into the claim (and getting information from all sources including the invigilator/ exam centre/ tutor/ etc.) The result of this behaviour is that staff will turn a blind eye. It's not worth the trouble and stress of reporting when the Dean dismisses it without the students even getting a warning!
Emphasize and communicate the importance of plagiarism/ academic integrity across the university		If faculty do not take an active role in educating their studetns [*sic*] about academic integrity, and follow through with penalties in cases of academic dishonesty, students will not take the issue seriously and/or remain ignorant about the definition of plagiarism
	Encourage faculty and tutors to be vigilant about plagiarism, contract cheating, and other instances of academic misconduct	vigilance is the only method for deterring cheating and plagiarism. If we don’t recognize it then we can’t intervene. This has become a larger job with the easy access of the internet but if it is easy for students it is easy for us to keep on top of it I have contacted my tutor about my concerns about academic integrity and I have been assured that proper safeguards in place
	Encourage students to value academic integrity and learning through extrinsic as well as intrinsic motivational strategies	I am paying for my own education and will not benefit if I cheat By cheating you’re just showing you don’t know the material, meaning you won’t know it when you’re in the work force Student participation should mainly involve communicating distaste for cheating in all its forms and thus influencing the culture of [the university]
	Appeal to students’ maturity to remind them of their responsibility in engaging in academic integrity	I would guess that a majority of students go to … University because they really want to better themselves. In such cases, cheating would serve no purpose
Be compliant with policy (all instructors need to do this)		I think it should be clearly stated as part of our jobs and emphasized as such I am confident in [this] University and while I have not been involved in any direct occurances [*sic*] around cheating I am confident that [this] University faculty would handle them effectively according to policies
Encourage instructors to model academic integrity	Keep in mind that instructors serve as models in their writing and teaching, so they should ensure that they cite sources properly in course materials	I think the faculty role is crucial; if we do not promote academic integrity, students will probably assume it is not important. This goes beyond cheating and plagiarism to taking responsibility for poor work and pride in good work. Faculty have to model integrity in their assessment of student work and in dealing with grade appeals, i.e. not granting requests for grade increases without good reason but doing so when we have been unfair
	Inform students of instructor expectations regarding academic misconduct	My instructors did not really discuss anything of this nature with me. The information was simply posted in each course
	Consider embedding academic skills into each course, using online technologies to deliver and scaffold	-there’s not enough thought given to information literacy and skills in curriculum planning -training in information literacy, research and avoiding plagiarism needs to be worked into every course -instructors need to model academic integrity in their lessons and in the texts they choose

Our participants were not alone in feeling unsupported by administration. The literature reports perceptions of inconsistent application of policy (Chugh et al., 2021) and lack of follow through by administrators (MacLeod & Eaton, 2020). Instructor fears of not being supported seem to be justified, as even 20 years ago, Whitley and Keith-Spiegel (2001) reported “horror stories abound about administrators who abandon instructors who have the courage to pursue cases of academic dishonesty” (p. 334). Commitment from administration is critical because, as our participants’ comments illustrate, personnel may stop reporting cases of misconduct if they do not feel supported (de Maio et al., 2019).

A reason for inconsistency and non-compliance may be a lack of shared understanding of what constitutes misconduct. Another reason is fear of lawsuits from dissatisfied students (Amigud & Pell, 2021a). This was not mentioned often, but it did arise.

Many instructor respondents, like those described in the literature (Amigud & Pell, 2021b), took it upon themselves to deal with cases of academic misconduct. As one faculty member noted, “there isn’t consistency. In addition, although my tutors have been informed about the policy, many don’t comply, thinking it easier to assign their own consequences. In short, then, non-compliance is a huge issue.” Lynch et al. (2021) reported that the complexity and difficulty of reporting and following through on misconduct cases contributed to lack of compliance by instructors. As well, inconsistent application of policy and procedures may confuse stakeholders about what is acceptable and what is not. Lack of clarity can result in instructors applying differing standards, which is perplexing for students (Gottardello & Karabag, 2020). Respondents suggested that differing standards in administrative application of policy can confound instructors as well.

The literature confirms there are individual interpretations of policy as well as distinct ideas as to what constitutes academic misconduct. Researchers have verified that students receive mixed messages from different instructors even within their institution about what is acceptable writing behaviour (Sutherland-Smith, 2018), and what constitutes collusion (Bertram Gallant, 2008).

A final topic raised by respondents was the role instructors could play in supporting and modelling academic integrity, going beyond issues of misconduct. Gottardello and Karabag’s (2020) multinational study revealed that some faculty felt it important to serve as “leaders” (p. 12) in academic integrity, acting as role models as well as motivating and coaching students to be their best. Participants in our study expressed similar sentiments.

Many researchers and practitioners advocate replacing or supplementing traditional punitive academic integrity policies with educational and restorative justice approaches (Benson et al., 2019; Sopcak & Hood, 2022). They acknowledge acts of academic misconduct are not simply moral issues but often issues of development of writing or other skills (Jamieson & Moore Howard, 2019). However, there is wide variation in instructor engagement with academic integrity and not all instructors feel that teaching about it is their responsibility or are willing to take time away from other aspects of their workload to do so (Peters et al., 2019). Hunter and Kier (2022) proposed that administration could demonstrate commitment to academic integrity by providing required resources. We turn to the topic of resources next.

### Resources

We identified 45 comments that spoke to the need for resources to address three key concerns: (1) compensation for pursuing alleged cases of misconduct, (2) education and training that promote academic integrity, and (3) effective course design. Six recommendations and 19 strategies articulated the need for appropriate resources and support for faculty, administration, and students. Faculty and tutors complained about the extra time and work involved in and inadequate compensation for seeking and providing sufficient evidence for investigations (Table 4).

**Table 4:** Selected Recommendations, Strategies, and Responses for Resources

Recommendations	Strategies	Responses
Provide resources and support for:		
Pursuing alleged cases (especially for Tutors/Academic Experts)	Compensate tutors for the extra work involved in ensuring academic integrity (e.g., to grade re-written assignments)	The primary reason tutors don’t catch plagiarism is that they are too busy, and following up on it creates too much hassle. Easier to pass the student than to do all the paperwork and follow up required
Promoting academic integrity	Educate and discuss with faculty members what measures are available for increasing academic integrity. Obtain their viewpoints about what they think is needed and what they might be willing to do. Have some sort of interaction among faculty and/or allow access to other faculties’ Moodle sites to generate and exchange ideas about creating exams designed to minimize cheating	I believe review of existing support areas i.e. tutors, etc. need review to determine opportunities of improvement to better support students who may struggle with certain courses, assignments, tests
Educating faculty/tutors to understand the importance of course design	Change assignments and exam questions frequently	Most important is proper course, assignment and exam design I could change assingments [*sic*] more often but this is slow process, and it takes time to have them changed. The same with exams
Creating solid course, assignment, and exam design	Create unique and personal assignments Consider group work design that increases the chances that each student contributes equally	It is not necessary to have closed book exams and tests I’m also ambivalent about throwing certain types of group work on assignments into the plagiarism bucket–peers can contribute a great deal to genuine learning
Ensure that assignments/exams are interesting, meaningful, and relevant (to students’ lives)		Greater diversity in assessment would … make the assessments more interesting, meaningful and relevant and hopefully create less likelihood of cheating ensure assignments require students to conduct original and very specific material that cannot be duplicated

The literature reports similar concerns. McKay (2014) described the detection and enforcement work of combatting plagiarism as time and labour intensive. Whitley and Keith-Spiegel (2001) identified elements of an effective academic integrity program as formal administration, a committee, communication, and training for both faculty and students, implying a need for time and assistance for both faculty and administrators. Workshops for academics, administrators, and other post-secondary staff in Australia were found to be successful in increasing understanding, knowledge, and practical ideas for encouraging academic integrity (Curtis et al., 2021), so support for such training seems essential.

Our respondents recommended faculty input into and responsibility for academic integrity, highlighting their need for resources to enhance student learning. Similarly, Lynch et al. (2021) called for more resources from the institution, such as lighter workload. (This would also provide time for faculty to be involved with drafting policy). These researchers also requested more institutional support for educating students about academic integrity. Creating effective institution-wide tutorials such as those by Benson et al. (2019) requires heavy resources.

The instructor role of promoting and modelling academic integrity (Bristor & Burke, 2016) requires resources to encourage student learning through education, information, and course design (Gottardello & Karabag, 2020). Course design is an important factor in enhancing faculty understanding of how to support academic integrity. Working with a learning designer can help in this process. Understanding emerges from professional development workshops or presentations and informal conversations about academic integrity. Course creators also learn from the process of designing specific course-based strategies that support student learning and performance integrity.

For example, instructors can learn to design their courses so that they include frequent assessment redesign as a way to prevent cheating (Eaton, 2017). Our respondents noted, however, that while this may be an ideal strategy, the implications of time, resources, workload, and complexity need to be considered. Using online examinations is an example: there are a number of people involved in setting up successful and secure testing – course production staff, faculty, registrarial staff, and others. Furthermore, cheating can happen even when using a proctor (Samela & Martin, 2021). Similarly, some respondents suggested alternative assessments could remove much opportunity for cheating through enhancing flexibility and meaningfulness. Research demonstrates creating unique and personal assignments that request students’ thoughts and ideas results in opportunities for learning (Conrad & Openo, 2018; Lang, 2013), beyond misconduct avoidance.

Respondents stated course design should include “online lessons in academic skills” such as information literacy and academic integrity, supported by quizzes for self-testing, opportunities to practice appropriate paraphrasing and citation, and in-course discussions. The literature includes examples of this (Bates, 2019; Mackay, 2014). McDonald and Adl (2019) pointed out that “students learn best how to choose, use, and document through sources, through experiential learning, that is, by working with quotes, and bibliographic forms in authentic, context-based exercises” (p. 95).

Recommendations for reconsidered assessment methods included using rubrics that clearly describe the purpose of assignments and the distribution of weight for various elements of the work, and minimizing the grade value for assignments that could be used for cheating. Some of these suggestions fit recommendations from research and instructional design practice (see for example, Conrad & Openo, 2018; Laurillard, 2012; Melrose et al., 2013). Suggestions for declarations of integrity align with Lang’s (2013) observation that signing an honour code just before taking an exam increased academic honesty. As well, respondents suggested group activities be redesigned so they appreciate the contributions of peers and increase the chances that each student contributes equally.

Several resondents made requests for text-matching software. A common assumption is that this is a panacea for detecting plagiarism (Mphahlele & McKenna, 2019) or for correcting students’ papers (Bailey & Challen, 2014). However, while some researchers encourage using text-matching software (Graham-Matheson & Starr, 2013), others contend there are contraindications for using it (Weber-Wulff, 2015). Shifting the focus from plagiarism detection to prevention prioritizes academic literacy (Clark et al., 2020) and deserves further research.

## Discussion

Our survey asked how the university could improve policies on academic integrity, how faculty and tutors handled cases of misconduct, about satisfaction with how academic violations were treated, and about the role of faculty and tutors in encouraging academic integrity. This paper focuses on the responses to seven open-ended questions that collected suggestions from students, faculty, and tutors for reducing cheating, minimizing academic misconduct, and other general perspectives about academic integrity. We analysed the comments, looking for patterns, consistencies, and contradictions, with a view to identifying recommendations for enhancing academic integrity in the university. Our analysis was conducted in multiple iterations over several months and involved discussion, visualization, and co-writing to explore our interpretations of the meanings of the various perspectives expressed in the responses. In addition, we received support for our ideas from members of our academic integrity community through presentations and discussions.

The depth of comments and the wide range of recommendations that emerged provide a strong basis for us to represent these ideas as suggestions for strengthening academic integrity practice. Respondents spoke at multiple levels. Many referred to what individuals can do, while others offered comments related to course or program design or potential administrative actions. Others recommended an approach to institutional level support for academic integrity grounded in an appreciation of people’s opinions, attitudes, and experiences, that can lead to enhanced policy and procedures that are clear, consistent, and implementable. We infer that stakeholders might be more committed to complying if their input were invited and respected. Policy that provides for adequate, appropriate resources would also help to ensure compliance and commitment are possible.

This section discusses the key issues of concern we identified in participants’ comments and that are addressed in the literature. We observed perspectives on reducing academic misconduct as well as for enhancing integrity. Both the responses and the literature advocate a coordinated approach among many strategies. Students and instructors benefit from alignment between principles and practices and from participation in the policy development process. As Thacker and McKenzie (2022) assert, a holistic strategy can ensure a culture of academic integrity throughout the institution. Each individual and stakeholder group has a role to play for this to happen.

Institutional administrators are responsible for creating and disseminating clear policy and procedures. Providing education and professional development assures that students, instructors, and staff are aware of policy and procedures related to cheating, plagiarism, and reporting academic misconduct – especially who to contact, how, when, and what evidence to provide. It is important that the range of possible consequences is appropriate to the nature of offenses and that stakeholders are aware of the variations among them. Administrator accountability includes budgeting for resources and assistance for pursuing alleged cases, and for ensuring instructors and staff have the skills needed. Adminstrative championship of academic integrity is essential. Without leadership commitment there will be little compliance from the rest of the institution (Bertram Gallant & Drinan, 2008). Multiple respondents stressed that compliance and commitment, including taking potential misconduct cases seriously, need to be demonstrated by administrators as well as instructors and students.

Instructors should keep in mind that they are role models in their writing and teaching. Faculty demonstrate their commitment by changing assessments frequently, and ensuring that they are interesting, meaningful, and relevant to students’ lives. Instructors are expected to comply with academic integrity policy and to follow stated procedures, but must feel competent and supported to do so. Ensuring that students understand and observe the guidelines for effective academic writing and avoiding misconduct is evidence of commitment. Their diligence in these tasks should be rewarded rather than dismissed by administrators.

Students in our study acknowledged they had a role to play in supporting academic integrity. Some suggested that they monitor and report one another, but others thought this could lead to rejection by fellow students. Several noted that each individual was responsible for their own conduct.

The operationalization of all these responsibilities requires resources. While detection of academic misconduct and punitive approaches are labour intensive and time consuming, so are prevention strategies and educational initiatives. Respondents’ comments confirm that adequate compensation for instructors is necessary to ensure that investment in policy development is balanced by investment in implementation strategies.

### Limitations and considerations for future research

Our study was limited in several ways, each suggesting opportunities for further research. First, at the time we conducted this survey, we focused on academic misconduct rather than academic integrity. We did not define academic integrity. Rather, we asked questions about and provided examples of “questionable behaviours”, “cheating”, “plagiarism” and “academic misconduct”. In our invitation letter, however, we identified the benefits to participants as enabling us to “help people (students, faculty and administrators) to place more emphasis on academic integrity and improve institutional policies and practices”. We anticipated that principles for effective assignment design, guidelines for new policy development, and enhanced understanding of stakeholders’ perspectives would emerge despite the lack of any definition. Further studies that are explicit about definitions and aspects of academic integrity may help uncover additional perspectives about the concept, as well as about misconduct prevention.

Second, although we received many comments about what administrators could and should do, we did not approach administrators themselves for their perceptions. Their contributions likely would have provided different perspectives from the students, faculty, and tutors we surveyed. Although we use the term administrators with respect to their assumed responsibilities, we were not always able to identify positions such as Academic Integrity Officer, Program Director, Dean, Registrar, or other staff. We acknowledge that each of these roles has different responsibilities, and it may be inappropriate to refer to them as if they were all the same. Future explorations of the beliefs and practices of various administrators may lead to enhanced understanding of issues. As well, we noticed in the comments certain expressions of adversarial positions between faculty/ tutors and administration. We do not know whether administrators share this attitude. It would have been helpful to have been able to triangulate stakeholder perspectives, but this remains an item for future research.

Third, we are reporting on the perspectives of students, faculty, and tutors from one Canadian online university at a particular moment in time. We realize that comments and interpretations may not be generalizable to other higher education environments, whether online or not. While we have shown evidence of literature that supports many of the key issues of concern at our university, more studies that explore academic integrity from multiple perspectives could replicate and build on our contributions.

A final limitation, which might also be seen as a strength of our study, is that the researchers come from different backgrounds. One author is more acquainted with qualitative research designs and experienced a variety of university roles including administration and faculty. She is experienced in teaching, applying, and writing about course design. The other author is more experienced with quantitative methods and more directly connected to the experience of students, faculty, and tutors. She has published on accidental plagiarism from a pedagogical perspective. Perhaps because of these differences, we were challenged to determine whether one aspect of the recommendations is more essential than the others, or whether there is an implied hierarchy. This may be a question for future research. Our differences made our analysis and interpretations more complex and fulsome. We used these differences as strengths to ensure we were as comprehensive and exhaustive with the data as possible. We feel confident that we conducted a thorough and balanced study that will be of benefit to current practitioners and future researchers. We believe we engaged in “interthinking” during the process of interpretation and writing, which is “how people, through joint intellectual activity, can use language to think together, make sense of experiences and solve problems to achieve more by working together than alone” (Omland, 2021, p. 4). We suggest future researchers attempt similar collaborative approaches to interdisciplinary explorations.

## Conclusion

In line with our findings and interpretations, we encourage a balanced strategy for supporting academic integrity that considers the three key areas of concern we identified. We learned that Policy and Procedures, Compliance and Commitment, and Resources are interdependent. Participants suggested failing to achieve balance weakens overall academic integrity. For example, for faculty to comply, they depend on resources and support for their commitment, and on guidelines provided, while administrators may tend to focus on quasi-legal aspects of policy and procedures (McDonald & Adl, 2019). A strong academic integrity culture can address these imbalances in priority and action. This may be accomplished through conversations that bring differing perspectives together. Our conclusion about shared responsibility aligns with current research emphasizing the importance of collaboration amongst all stakeholders (Miron et al., 2021; Packalen & Rowbotham, 2022).

Our project expands on previous research about policy and procedures (Bretag et al., 2011; Stoesz & Eaton, 2020) by including the areas of compliance and commitment and resources as essential to enacting effective policy. Without attention to the strategic and practical considerations of implementation, academic integrity will be insufficiently supported. Given the number of comments requesting education for instructors and students, our work is consistent with literature that calls for interventions supporting prevention of academic misconduct (Benson et al., 2019; Curtis et al., 2021). This may be especially important since the pandemic alerted many institutions about the need for enhancing academic integrity.
